# Monthly Summary

**Published:** 1894-12

**Authors:** 


### Monthly Summary.

LITTLE AIDS IN PRACTICE.—Perhaps one of the safest methods of annealing sheet gold is upon a piece of mica, when the flame cannot possibly touch the gold. This method works admirably with most makes, but I find that some varieties of soft gold seem to require additional heat, and this too, by direct contact with the flame, after it has been made up ready for use.

To carry very soft mixed cement to the bottom of a cavity, remove it from the spatula on to a wee bit of paper laying at the edge of the glass slide; then, with tweezers, carry this edge-wise up between the two teeth, if an approximal cavity, and press home with an excavator.

Miniature napkins cut or torn from pure white old cotton are preferable to those from new material, and should always be thrown away when once used. I trust none of us have the heart of a certain western operator, who compelled his better-half to wash and iron an innumerable number of these requisites. One of these folded up and laid between the lip and gum, or under the tongue, wonderfully extends our time in brief operations, and makes it quite possible to perform dry work. When removing debris from a cavity, one of these little squares held in the left hand, ready to take the refuse from about the excavator is but another item of carefulness and consideration on the part of the dentist that elevates him in the estimation of the patient, in fact, the need for these grows so rapidly that we soon find ourselves well supplied with them. Not infrequently persons drop in to have one or more of the incisors ground down to match the rest; to expedite the work hold a wet sponge against the wheel, and then you can work right along without interruption.

An especially convenient chisel is one rather broad, and the end-cutting edge ground out fish-tailed. The two sides may also be brought to a levelled, sharp edge. Try it.

Shave down silver nitrate and dust this over a freshly exposed pulp, when the patient is enduring great pain and will not tolerate the usual application at once.

When applying the cord, and it persists in slipping back into the cavity, make the knot secure, then carefully push up both it and the dam to position, when it will usually remain.

I have much faith in, and therefore disinfect every cavity immediately before filling.

Keep an oiled wool cloth to finish cleaning instruments, and they will always remain bright.

For use under anæsthetics, one pair of universal forceps should be a part of every set, in fact, these are never to be despised, if well made, and with curving beaks so to pass well over all crowns and bring the pressure well up on neck of the tooth. I am especially successful with those weak and broken down lower molars, frequently so trying, by taking hold well down or cutting through on to the anterior root, which is the curving one if at all so.

With certain varieties of flat mouths, score the model close up and around the air chamber, also several more across those soft portions of the palate, usually found on either side of the median line and back from the chamber. When smoothly done, these raises on the palate surface of plate do not seem to irritate the mucous membrane.

Occasionally a sharp, deep groove extends back along the same median line, use soft palate rubber over this.—*Dominion Dental Journal.*

HINTS.—By W. A. Brownlee, M. D. S.—If the rubber dam is punctured during the process of filling, dry it thoroughly and touch the spot with a thick solution of chlora-percha, such as is used for nerve canals, and allow the chloroform to evaporate before proceeding with the work. This will effectually exclude the moisture.

If you are troubled with the top plate of the flask coming off while packing, drill two holes near the rear end of the plate about half an inch apart, make a loop of heavy brass wire, insert in these holes from the inside and rivet in place. If the loop projects half an inch into the plaster it will not interfere with the denture, and will save you considerable annoyance.

Instead of soldering small strips of gold to clasps to retain them in position on rubber plates, use two platinum pins from a broken porcelain tooth. The clasp is held firmly to the plate, and the plate is not so liable to crack as if the strips are used.

A convenient rubber heater is made by taking a piece of asbestos an eighth of an inch thick and eight inches square, put a tin rim on it to strengthen it, dip it in water, wipe the surface, lay the rubber, ready cut, upon it, and place over the vulcanizing lamp for a minute or two before packing.

Dark joints, in repairing a denture made with gum sections, are produced by the charring or burning of particles of food, etc., with which the joints have become filled. This cannot be prevented unless the sections are removed from the plate and the adjacent ends dressed on the wheel.

When filling a number of cavities with gold at the same sitting, put on the rubber dam before you begin to prepare the cavities; remove the rough decay from all, then complete the preparation of the one you suppose will give the least pain, and fill it. By the time you have it completed the other cavities will be so thoroughly dried out that they can be excavated almost without pain. Try it.—*Dominion Dental Journal.*

THERE was a nice appearing lady dentist at the last New Jersey Convention. Introducing herself to me, she asked me into a side room to drink with her. I assured her I drank no intoxicants. Then she took from her side pocket some cigars and said to me and her lady companion that just then came up, "Then will you not smoke with us?" I was shocked. "Is this the proper thing to do in your community?" said I. They both assured me it was quite fashionable, and therefore, of course, quite proper, both to drink intoxicants in moderation and to smoke tobacco; and that this side room they invited me into was the headquarters of the officers of the society. At the hotel where we met in session there was no bar, so the officers had improvised one, for there were no saloons in the city. And what these ladies thought was more delightful was that the liquor and cigars were free.

Oh, I have misstated one thing. These were not nice appearing young ladies that invited me into this side room to drink with them; they were nice appearing young men. But why should this make much difference? Why should we have one standard of morals for the young men and another and higher one for the young ladies? Why should we men feel at liberty to do what we would not approve in our wife and mother? How coarse and vulgar it would seem to see a smoking, drinking lady dentist! We would say she was no lady, and she would have few customers of the better class.—EDITORIAL in *Items of Interest*.

---

DISEASE OF THE ANTRUM.—By Dr. Talbot.—The antrum cannot be depended on as to size, that is, whether it is as large as one's little finger or as a hen's egg, and extends over to the roof of the mouth, cannot be determined from appearances. We have seen skulls from which it would have been impossible to extract a tooth on one side of the mouth without going into the floor of the nose. We have reached the conclusion that the point between the second bicuspid and first permanent molar was the proper place to enter the antrum in operating. You must not lose sight of the septum which divides the antrum into two chambers. If you go up into only one antrum, the patient's head should be laid first on one side, then on the other, so as to drain both antra thoroughly. Treatment after opening should be simple. Cleanse out with syringe, using warm water or a weak solution of listerin or boracic acid, or something which will not injure the mucous surface. Many specialists use too strong remedies. Very simple treatment only is needed; cleanse thoroughly with warm water, and let nature do the rest.

In disease of the antrum the dentist should take entire charge of the case, which he should be competent to treat. We do not consider a man a real dentist who cannot do so, though we fear there are comparatively few who are qualified; and we should not attempt to open into the antrum without knowing that it was necessary. We might go so far as to extract a tooth, but should then wait two or three months, if there was any doubt, to give time for a cure of the trouble by nature's help. Then, when sure that it was required, we should make the opening. A drainage tube is not always necessary. If properly cleansed, the orifice made will sometimes heal up in twenty-four hours. In mild cases of antral trouble, where transmitted light gives no indication, we should treat the patient constitutionally. Ninety-nine out of one hundred cases are due to inflammation of the mucous membrane, as from the effects of grip or the inhalation of extremely cold air.—*Dental Cosmos.*

---

COMPARISON OF PROFESSIONAL FEES.—The *Pacific Medical Journal* in the course of an able editorial, says the following :

Why a lawyer should be paid 500 per cent. more than a doctor for doing five hundred times as little work, we suppose is entirely owing to the fact that the lawyer is that much more capable of taking care of his own interests. Exactly why the secular press of this country should take the same view of the case is a mystery. A case in point. The daily papers are congratulating ex-President Harrison on receiving a fee of $25,000 for four hours' work in court; had a medical man of equal ability as Mr. Harrison charged a many times millionaire $5,000 for a month's constant attention, the whole press would be charging him with robbery—a man to be avoided when you are sick, etc. Another case in point. Judge Levy, of this city, has just allowed a firm of attorneys a fee of $80,000 for looking after the routine business of an estate for a few months, and yet this very same judge refused to allow a fee of $30,000 which a medical man had presented for many months' attendance on a millionaire and his family. The actual work was probably one hundred times more than that performed by the attorney who received $80,000; while the responsibility was probably five hundred times more, yet his Honor, Judge Levy, saw fit to cut the doctor's fee down to $10,000. And why?—*Med. World.*

---

THE CONTAGION OF THE COMMUNION CUP.—Dr. Albert S. Ashmead, of New York, recently wrote a letter to the *Sun* of that city, worded as follows:

"The last time I knelt at the communion altar of the Episcopal Church, there knelt at one side of me a patient whom I knew, as I was treating him at the time, to be a syphilitic; his mouth had mucous patches, which make the disease especially contagious. This person took the cup before it came to me. Of course, I let the cup pass. At another time, the person next to me, but following me in the use of the cup, was also a patient of mine, in an advanced stage of tuberculosis. The mouth of this person was in a condition dangerous to his neighbor.

"Of course, no man who is not a complete survival of the Middle Ages can assert that, under these circumstances, a man (if he knew) would apply his lips to a probably dangerously contaminated cup, trusting in the protection of the Lord, who has allowed hundreds, a hundred times, to perish in burning or earth-shaken churches, while they were in the very act of worshipping Him."

The *Sun*, in a subsequent issue, thus editorially commented on these facts, when it said:

"When a physician writes to a newspaper, as one wrote to the *Sun* the other day, that he refrained from drinking from the chalice at a recent celebration of the Eucharist in the Episcopal Church because before him two patients of his had sipped from the cup, the one afflicted with an odious and the other with a destructive communicable disease, reasonable fears of the danger of the hallowed practice are excited even among the most devout of communicants; and such fears, thus justified, are quickly and widely diffused. It is unlikely, then —is it not probable—that this alarm will become so extensive and so great that all churches whose doctrines require that both elements shall be administered to the whole body of communicants will be compelled to imitate the Rochester example, or adopt some other method devised for the same purpose, in order to save the communion in both kinds from perilious disease?

The time is rapidly approaching when public attention will be more generally directed to this constant menace to health.—*College and Clinical Record.*

---

DIED WHILE HAVING A TOOTH EXTRACTED.—A negro woman recently died in Macon, Ga., while having a tooth extracted. The woman had been suffering severely from an aching tooth, and to get relief her husband called in a physician. It had been the custom of this doctor to administer chloral to lessen the pain in extracting teeth, and in this case he gave the woman fourteen grains. While waiting for it to take effect he visited another patient, and on his return found the woman stupefied. With the assistance of the husband he then proceeded to extract one of the teeth. While extracting it the woman struggled and screamed. The husband pointed out to the doctor another offending tooth, and insisted on its extraction. She resisted and struggled, but the husband held her while the doctor extracted it. She became quiet, but it was soon discovered she was dead.—*Dental Luminary.*

ALCOHOL IN SHOCK.—Dr. Wood is authority for the statement that "alcohol is probably of no value whatever in shock. Indeed, I am perfectly sure that a large dose of alcohol in shock puts one nail in the coffin of the patient, and if you want your patients to come out of shock you will be very careful in giving them alcohol. Alcohol stimulates the heart but paralyzes rather than stimulates blood-vessels." The theory is, "by its action on the blood-cells it checks oxidation and limits the power of absorbing oxygen and eliminating carbolic-acid gas."—*Omaha Clinic.*

---

HYDROGEN DIOXIDE.—$H_2 O_2$.—BY L. D. KASTENBINE, A. M., M. D., Professor Chemistry, Urinology, and Medical Jurisprudence, Louisville Medical College; Professor Chemistry Louisville College Pharmacy.

This remarkable liquid which contains the greatest percentage of oxygen of any compound known, was, for sometime, considered as a mere solution of oxygen in water, and consequently was called oxygenated water. It was afterward obtained free from water and found to be a definite chemical compound of hydrogen and oxygen, and differing from water in containing twice as much oxygen. In this state it is a heavy, oily liquid, readily decomposing at ordinary temperatures, but if heated, with explosive violence, being converted into ordinary water and oxygen gas. When poured into water it sinks, being nearly half again as heavy as that liquid, but is miscible in all proportions with it. It has a somewhat bitter, astringent taste, and is colorless, transparent and without odor. It contains 94 per cent. of oxygen gas by weight, and will yield 475 times its volume of that gas. It bleaches the skin, hair, ivory and destroys organic coloring matter, pus and all organisms with which it comes in contact by liberating oxygen gas in a nascent or active state. It is resolved into oxygen and water by certain metals, such as gold, platinum, silver and mercury in a state of fine subdivision, although the metals themselves undergo no change whatever. If the oxides of these same metals are brought into contact with it, not only does the hydrogen dioxide lose oxygen and become water, but the oxides lose their oxygen and are reduced to the metallic state, thereby evolving an additional amount of oxygen.

Strange as it may appear, with all its energetic oxidizing action, it has no effect on phosphorus, a substance which is so readily oxidized by the air.

The preparations found in commerce are only solutions of this compound in water, and sold in different degrees of concentration or strength, rated by the number of volumes of oxygen gas they can be made to yield. A fifteen volume solution is one that will give off fifteen volumes of gas from one volume of the solution. A ten volume solution will yield ten pints of oxygen gas from one pint of the solution, and so on.

These solutions, although more stable than mere concentrated preparations, nevertheless decompose and lose their nascent oxygen on which its powerful antiseptic powers depend, and consequently we find the commercial brands varying considerably from their reputed strengths. The solution I find containing the greatest percentage of available oxygen, is the preparation known as Marchand's, which when perfectly fresh, is about a fifteen volume solution.

There are quite a number of different methods of preparing aqueous solutions of this interesting compound besides the original method of Thenard, the discoverer. Usually, however, barium dioxide in the hydrated state and purified from all foreign matter, is decomposed by such acids as will make an insoluble compound with it. The United States Pharmacopœia has adopted this compound under the official title of Aqua Hydrogenii Dioxidi, gives a process of preparing it and describes it as a slightly acid aqueous solution of hydrogen dioxide, containing, when freshly made, about 3 per cent. by weight of the pure anhydrous dioxide, corresponding to about 10 volumes of available oxygen. It is made by the action of phosphoric acid upon barium peroxide. It must be born in mind that it is essential to employ a small amount of free acid to preserve these solutions, but if too large a quantity it would be a source of irritation when applied to denuded surfaces and inflamed mucous membranes, and consequently, officially, a preparation requiring more than 0.5 c. c. of volumetric caustic potash solution to neutralize .50 c. c. of it, does not come up to the U. S. P. standard.

Of the various brands of commercial dioxides I have examined, I find Marchand's to be the one which yields the largest amount of available oxygen under all conditions of exposure, and the one which contains the minimum percentage of free acid. All the marketable articles I have seen are free from barium compounds, but the majority do not come up to the 15 volume standard, but are 6, 8, 10 and 12 volume solutions.

In addition to its medical uses, hydrogen dioxide can be employed to detect blood, in conjunction with freshly prepared tincture of guaiac. Although tincture of guaiac turns blue with a variety of substances, blood is not one of them. So in testing for a stain—say on clothing—moisten the spot with water, and afterwards apply a piece of white filter paper; the slightest straw-colored stain on the paper suffices. Now, add to the spot on the paper a few drops of the guaiac tincture—no coloration. Add a few drops of solution of peroxide, when instantly the spot turns of a deep azure blue. Of course if the spot turns blue by the guaiac alone, it can not be due to blood, yet it is possible blood may be present with some other substance which has that property, and hence the employment of peroxide, in that case, would be a source of fallacy. If there is no bluing by guaiac and peroxide together, then absolutely no blood is present.

Hydrogen dioxide can be determined quantitatively by permanganate of potassium solution acidified by sulphuric acid, and the quantity of oxygen gas evolved measured in an instrument called a nitro-meter, and calculated for normal pressure and temperature. One-half the oxygen evolved comes from the dioxide and the other half from the permanganate solution.

Another method, and the one commonly employed, is to add a volumetric solution of permanganate of potassium from a burette to a measured portion of the hydrogen dioxide solution, diluted with water and acidulated with sulphuric acid, until the permanganate solution is rendered colorless, and then a few drops more of that reagent employed till a permanent faint pink coloration is given to the dioxide solution to indicate the completion of process. A slight calculation will give the strength of solntion. There are other methods, but the two indicated are the best.

A solution of peroxide of hydrogen is usually tested by pouring a drachm of it in a clean test tube, together with an equal quantity of ether, then pouring into the tube a few drops of bichromate of potassium solution, and shaking the tube, when the ethereal layer will become of a beautiful azure blue color, due to the formation of perchromic acid which dissolves in the ether.

To a few drops of nitrate of silver solution, add aqua ammonia enough to precipitate the oxide of silver, then add hydrogen peroxide when finely divided metallic silver separates. A solution of titanic acid in oil of vitrol and diluted will yield a yellow color wh n added to solutions of the peroxide.—*Louisville Medical Monthly*, July, 1894.

